# Medication Error Reporting: Underreporting and Acceptability of Smartphone Application for Reporting among Health Care Professionals in Perak, Malaysia

**DOI:** 10.7759/cureus.2746

**Published:** 2018-06-05

**Authors:** Doris George, Amar-Singh HSS, Azmi Hassali

**Affiliations:** 1 School of Pharmaceutical Sciences, Universiti Sains Malaysia; 2 Department of Paediatrics and Clinical Research Center Perak, Raja Permaisuri Bainun Hospital Ipoh, Ipoh, MYS

**Keywords:** survey, medication error reporting, smartphone, under-reporting, doctors, pharmacists

## Abstract

Background and objectives

In Malaysia, the national voluntary non-punitive Medication Error Reporting System (MER-S) has been available since 2009, with compiled reports indicating the underreporting of various medication errors (ME). This survey intends to determine the ME reporting practice among healthcare professionals and the acceptance of ME reporting by utilising smartphone application if it is available.

Design

A cross-sectional survey was conducted for two months in 2017 among doctors and pharmacists in publicly funded healthcare facilities in Perak, Malaysia. The survey was distributed through various professional WhatsApp chat groups, and reminders were sent twice to the respondents.

Results

A total of 334 doctors and pharmacists responded to the survey; the majority were pharmacists (61.7%) with a median age (in years) of 32 (interquartile range (IQR) 29-36) and work experience (in years) of 7.5 years (IQR 5-11). The rate of respondents being aware of the MER-S and having encountered ME at the workplace was high, at 73.4% and 96.1%, respectively. However, only 44.8% reported using the system. The reason hindering them from reporting ME was primarily being in a busy and hectic work environment. Pharmacists were more likely to report ME compared to doctors (adjusted odds ratio (adj OR) 10.51; 95% Confidence interval (CI): 5.34, 20.6), especially pharmacists who had frequent encounters with ME at work (adj OR 2.84; 95% CI: 1.70, 4.81) and who perceived that ME can be handled well (adj OR 3.52; 95% CI: 1.93, 6.44). They were more likely to report ME. A majority (90.7%) had downloaded one or more digital medical applications to aid their work. The speed of Internet connectivity at the workplace was rated as “fast” or “good” among 136 (40.7%) respondents but among 130 (38.9%), it was “average.” The percentage of doctors and pharmacists that would report ME by utilising a smartphone application was 86.5% if one is available, and they preferred an application with a user-friendly interface, anonymity, and limited data-entry requirements.

Conclusion

Doctors and pharmacists were aware of MER-S and willing to report when they encountered ME. However, less than half of the respondents had used the system. With the primary concern of ME underreporting in a busy and hectic work environment, an alternative smartphone ME reporting application can be developed to complement the current MER-S considering that the respondents had positive responses to this method.

## Introduction

Patient safety is defined as the efforts taken to prevent errors and adverse events from reaching patients in the healthcare system [1]. The type of errors and adverse events can be further divided into administrative, communication, diagnostic, documentation, medication, surgical, procedural, and decision-making. Medication error (ME) is a subset of medical error and the most common type and can be actual or potential error or harm caused to patients due to failure in the treatment process [2]. ME include any errors in the treatment or medication process, including prescribing, dispensing, and administration.

The rate of prescribing error was reported as 8.8 (95% confidence interval (CI): 8.6-9.1) per 100 ordered medications [3], the administration rate was 8.0 (interquartile range (IQR): 5.1-10.9) per 100 total opportunities for error [4], and dispensing errors happened at a rate of 1.6 for every 100 prescriptions received [5]. In South-East Asia, the error rates for prescribing error, administration error and dispensing error were reported as 7%-35%, 15-88% and 14-35%, respectively [6].

Reporting ME is essential for learning from near misses as well as actual errors, thus preventing future errors [1] from occurring. In Malaysia, the national Medication Error Reporting System (MER-S), a voluntary and non-punitive system, was established in 2009 and thus ME reports increase annually [7]. However, the reports were mainly received from hospital pharmacists working in publicly funded healthcare facilities. The majority of submitted ME reports were related to the stage of the medication process, which is medication prescription involving near misses and medications that did not result in any harm to patients [7]. Encouraging ME reports from various healthcare professionals such as doctors and nurses, who are frequently involved in the medication process, allow a clearer picture of the actual medication error occurrences, thus improving the approach to prevent errors that are potentially harmful or even fatal.

In a qualitative study done in Malaysia, the reporting system itself was noted as one of the barriers to ME reporting. Another reason given by the respondents was that the reporting system does not guarantee confidentiality, is not simplified, and requires multiple reports [8].

Digital technology, such as smartphone applications, has been used in the medical world as a means of providing care for patients as well as education and a mode of consultation for healthcare providers [9-10]. Online versions of ME reporting have shown to improve reporting rates [11]. Handheld devices such as personal digital assistants (PDA) have been used to report ME and adverse events and have been found to be useful for healthcare professionals [12]. ME reporting using a smartphone application can also complement online ME reporting. An application is a software programme for computers or electronic mobile devices that has a distinct objective. ME reporting using a smartphone application provides the ease of having an ME reporting form in one’s own device and provides a platform for prompt user feedback. This study is a preliminary attempt to determine the acceptance of ME reporting using a smartphone application (app) as well as exploring current ME reporting practice and factors associated with ME reporting.

## Materials and methods

Design

A cross-sectional electronic survey involving doctors and pharmacists was conducted in Perak, Malaysia. Perak has the second largest official bed strength in the public sectors with 14 publicly funded hospitals, one institution and 85 health clinics [13]. The survey was conducted for two months (from August 2017 to September 2017) to achieve the required sample size.

Sample frame

A total of 1,590 registered doctors and 784 registered pharmacists were registered in Perak at the end of December 2016. Power calculations for population surveys were performed using the RaosoftÒ website (http://www.raosoft.com/samplesize.html). With the setting fixed at a margin of error of 5%, a confidence interval (CI) of 95%, and a response distribution of 60%, a minimum required sample size of 331 was obtained.

In Malaysia, medical graduates (both from local universities or overseas tertiary education) have to undergo a minimum of 24 months of compulsory work at approved publicly funded hospitals while pharmacy graduates require a minimum of 12 months of attachment at approved healthcare facilities to be fully registered before obtaining their license to practise. All registered doctors and pharmacists were included in this survey, and doctors and pharmacists undergoing their internship as mentioned above were excluded from participating in this survey.

Questionnaire development

The questionnaire was developed based on a literature review of the knowledge and practice of medication error reporting [14-15] and smartphone use [16-18] among doctors and pharmacists. The first part of the questionnaire required the doctors and pharmacists to state their demographics (age, gender, ethnicity-optional, work experiences, and work settings). The second part contained questions on the use of digital technology such as medical applications at work and internet accessibility. The final part contained questions on the knowledge of MER-S, frequency of ME encounters, reporting practices, and barriers to ME reporting and the acceptability of reporting ME using a smartphone application if one is available. The questionnaire was drafted and reviewed for face and content validity by two experts with more than 10 years of experience in patient safety research. The sequencing of questions was also restructured based on the feedback from experts. The electronic version of the questionnaire was distributed to five doctors and five pharmacists outside of Perak for pre-testing. The internal consistency was measured using Cronbach’s alpha, which was 0.814.

Distribution of questionnaire

The questionnaire was designed using Google Forms and a short hyperlink was created to access the survey (Appendix). This hyperlink was distributed into various healthcare WhatsApp groups such as a medical specialist group and a paediatric group, among others, for both doctors and pharmacists in publicly funded medical facilities in Perak. A lead member from each speciality group was contacted and asked to paste the hyperlink into their WhatsApp group. Lead members contacted were members of speciality group comprising of doctors or pharmacists in the state. The link was successfully distributed to at least 627 registered doctors and 532 registered pharmacists based on the distribution list provided by members of each lead members. If the respondents attempted the survey, it was considered consent to participate as the first page of the survey had a consent form attached.

Statistical analysis

The collected data were entered, cleaned, and analysed using IBM SPSS Statistics for Windows, version 20.0. (IBM Corporation, Armonk, NY, USA, released 2011). The data were presented in percentages and median and IQR. A chi-squared test was used to test the relationship between the respondent’s practice pattern and the severity of harm from the ME. Multiple logistic regression analyses were used to determine the association of five independent variables, profession, years of employment, work setting, frequency of encountering ME, and perceived handling of ME, with the dependent variable, reporters and non-reporters of ME.

First, a crude relationship between the ME reporters and each independent variable was conducted using a scatter plot and a simple logistic regression. Using automatic variable selection procedures, significant variables were identified by fitting all of the independent variables (regardless of their significance in a univariate analysis) into multiple logistic regression models. In this step, both forward and backward stepwise variable selection procedures were applied with p-value less than 0.05 considered significant and included in the model. All possible two-way interactions between the independent variables were also checked. The preliminary main-effect model was also assessed for multicollinearity by obtaining the variance inflation factor (VIF) for each independent variable. When the VIF was greater than 10, it was considered a significant multicollinearity problem and evaluated for model assumptions and outliers using residual plots. The model that met all of these required assumptions without outliers was then considered the “final model” and used to interpret the relationship between ME reporters and each significant independent variable. All hypotheses involved were two-sided tests and a p-value less than 0.05 was considered statistically significant.

Ethical approval

Ethical approval for conducting the survey was obtained from the Medical Review and Ethics Committee of the Ministry of Health of Malaysia. The registration ID for the survey is NMRR-15-1445-27125 (IIR).

Funding

This research received no specific grants from any funding agency in the public, commercial or non-profit sectors.

## Results

Respondents’ demographics

A total of 334 unique responses were received after the initial response and two reminders, resulting in a response rate of 28.8%. Their median age was 32 years (IQR 29-36), and their median years of service was 7.5 (IQR 5-11). Of the majority of the respondents, 209 (61.7%) were pharmacists and 223 (66.8%) were female. Table 1 summarises the characteristics of the respondents in further detail.

**Table 1:** Respondents’ demographics. IQR: Interquartile range

Characteristics	Total (%)	Median (IQR)
Professional category		
Doctors	128 (38.3)	
Pharmacists	206 (61.7)	
Gender		
Male	111 (33.2)	
Female	223 (66.8)	
Age (in years)		32 (36-29)
Years in service		7.5 (11-5)
Work settings		
Hospital	255 (76.3)	
Clinic	79 (23.7)	
Doctor, n = 128		
Medicine	32 (25.0)	
Paediatrics	26 (20.3)	
Surgery	19 (14.8)	
Primary Care	17 (13.2)	
Others	35 (27.3)	
Pharmacist, n = 206		
Outpatient pharmacy	88 (42.7)	
Clinical	42 (20.4)	
Inpatient pharmacy	32 (15.5)	
Procurement	15 (7.3)	
Drug information	12 (5.8)	
Others	17 (8.3)	

Current practice of medication error reporting

The overall percentage of doctors and pharmacists in the survey who had encountered ME at work was high at 96.1% but only 44.8% had experience with ME reporting. A majority of the respondents, 73.4%, acknowledged being aware of the national initiative for the MER-S in Malaysia, and 75% were pharmacists. Among the respondents, only 29.5% had ever received compilations on ME that were reported. The respondents who reported “no harm” were likely to report near misses (73.8%) compared to those who did not report “no harm” (59.4%) ($\chi$^2^(1) = 12.21, p < 0.01). Typically, the respondents who reported “no harm” were likely to report permanent harm or death (85.5%) compared to those who did not report “no harm” (69.8%) ($\chi$^2^(1) = 10.43, p = 0.01).

In the multivariate analysis of Table 2, the professional category, frequency of ME encountered at work, and perceived handling of ME by management were significantly associated with ME reporting at a 0.05 level. In the stepwise regression analysis, the pharmacists were 10 times more likely to report ME compared to the doctors (adjusted odds ratio (adj OR): 10.51; 95% confidence interval (CI): 5.34, 20.68). The doctors and pharmacists who encountered ME frequently (daily, weekly, or monthly) were three times more likely to report ME compared to those that encountered ME less often (adj OR: 2.84; 95% CI: 1.70, 4.81). Finally, the doctors and pharmacists who perceived that ME were handled well by their facilities’ governing board or management (rated as “excellent” or “good”) were 3.5 times as likely to report ME compared to those who perceived the handling of ME as “poor” (rated as “poor” or “not sure what was done”) (adj OR: 3.52, 95% CI: 1.93, 6.44).

**Table 2:** Results of logistic regression analysis. ME: Medication error; OR: Odds ratio by univariate logistic regression; 95% CI: 95% confidence interval; AOR: Adjusted odds ratio by multiple logistic regression adjusted for gender, profession category, years of service, frequency of ME encountered in practice and perceived handling of ME by management.

Variable	Univariate		Multivariate	
	OR (95% CI)	p-value	AOR (95% CI)	p-value
Gender				
Female	2.20 (1.35, 3.58)	0.001	1.23 (0.68, 2.23)	0.460
Male	1		1	
Profession				
Pharmacist	9.11 (5.10, 16.28)	<0.001	10.51 (5.34, 20.68)	<0.001
Doctor	1		1	
Years of service				
Less than 10 years	0.7 (0.44, 1.11)	0.123	0.85 (0.49, 1.47)	0.306
10 years or more	1		1	
Frequency of ME encountered in practise				
Frequent	3.38 (2.14, 5.33)	<0.001	2.84 (1.70, 4.81)	<0.001
Not frequent	1		1	
Perceived handling of ME by management				
Good	1.88 (1.18, 2.99)	0.008	3.52 (1.94 6.37)	<0.001
Poor	1		1	

When the respondents were asked about the main factors hindering them from reporting ME, having “no time” or being “busy” was the most frequent reason (41.6%), followed by worries of litigation issues (19.8%), concerns with reporting errors committed by colleagues (12.6%), fear of victimisation (9.8%), and reporting system design-related issues (9.3%). The reporting-related issues that hindered the reporting ME are summarised in Table 3.

**Table 3:** Respondents’ reasons for hindering reporting medication errors.

Reasons	Total (%)
No time or too busy	139 (41.6)
Legal implications	65 (19.5)
Concerns with reporting errors committed by colleague	42 (12.6)
Worried about victimisation	32 (9.6)
Impairs reputation	22 (6.6)
Complicated reporting process	13 (3.6)
No knowledge on reporting	6 (1.8)
No improvement seen	6 (1.8)
Necessary to perform a root cause analysis	3 (0.9)
Insufficient patient data	2 (0.6)
Not mandatory to report	2 (0.6)
Website errors	2 (0.6)

Acceptability of smartphone application for medication error reporting

More than half of the respondents, 62.9%, owned a smartphone with the Android operating system, one had a BlackBerry, and the remainder used the iPhone operating system (iOS). The majority (90.7%) had at least one medical application on their smartphones. The median number of medical applications downloaded was 3 (IQR 5-2), 63.4% of the respondents used the applications daily, and 22.1% used them on a weekly basis. The common medical applications downloaded by the respondents were medical references, drug references, and medical calculators. The top five medical applications downloaded were Medscape, My Blue Book, Micromedex, MIMS, and Lexicomp©.

When asked if they would use a smartphone application for ME reporting if one was available, an overwhelming majority of the doctors and pharmacists responded affirmatively, with only 45 declining. Smartphone application features for ME reporting that were considered the most important were user-friendly application (91.3%), anonymity of reporting (71.6%), and limited data-entry requirements (50.3%). Other suggested features included low memory usage, no lag time, security, the ability to generate reports, compatibility with most smartphones, and free notification of successful submission. The data above are presented in Table 4.

**Table 4:** Features of medication error reporting application for smartphones deemed important by respondents.

Features	Total (%)
User-friendly application	269 (92.8)
Confidentiality	210 (72.4)
Limited data usage	155 (53.4)
All of the above	128 (38.3)
Other	38 (11.4)
Other Features	
Simple form	9 (2.7)
Fast application	8 (2.4)
Provides reports or statistics	6 (1.8)
Secured	5 (1.5)
Ability to link to email or website	3 (0.9)
Notification of successful submission	2 (0.6)
Compatibility	1 (0.3)
Report traceability	1 (0.3)
Interactive platform	1 (0.3)
Good interface	1 (0.3)
Low memory usage	1 (0.3)

Only 16 of the respondents did not have access to the Internet on their smartphones at work. Their Internet access speed at work was reported as “good” by 40.7%, “average” by 38.9%, and “slow” by 15.6%. A total of 270 respondents used their personal data plan for Internet access on their smartphone at work, and 55 used employer-provided Internet access.

## Discussion

Questionnaire distribution via WhatsApp

Questionnaire distribution through Google Form using a hyperlink provided a fast and no-cost method of dissemination compared to the traditional postal mailing method. This link can be copied into emails and posted on other social networks such as Facebook and smartphone communication applications such as WhatsApp, Viber, and Line. The respondents’ responses were auto-generated into a spreadsheet, with the advantage of recording the time of the responses. This format was converted into statistical software for further analysis. This electronic method provided completeness of data by setting “required questions” for pertinent questions, added security, and ease of sending out reminders. The reminders increased the responses to complete the questionnaire; the responses surged by 14.1% and 18.3% with each given reminder, based on the time the reminders were sent out. WhatsApp was used to distribute the above questionnaire as it was cited as the most common messaging application used in Malaysia in a recent survey [19].

Knowledge of reporting system

In this survey, a high number of respondents knew about the MER-S, with the percentage of pharmacists higher compared to the doctors. This is likely due to the fact that the system is under the jurisdiction of the Pharmacy Division of the Ministry of Health of Malaysia. The Pharmacy Division conducts various workshops to promote the MER-S to healthcare professionals, particularly pharmacists. In various studies, a lower percentage of the respondents’ knowledge of the ME reporting system was reported in their workplaces compared to this research. Among physicians in the United States, only 54.8% knew about ME reporting [20]. In Australia, it was reported that although 94% of doctors were aware of the reporting system, only 43% knew where to obtain forms and 49% knew how to submit a completed form [21]. One study noted that the knowledge of the MER-S was low, ranging from approximately 28% to 31% of the respondents [15]. Some of the reasons cited for a lack of knowledge of ME reporting were improper implementation and lack of orientation in the use of the MER-S, non-permanent staff to handle the reports, and foreign healthcare professionals at certain institutions. The governing boards at certain healthcare facilities may have the impression that ME reporting education is sufficient; however, the healthcare staff may feel that further formal training is required [7, 22] for executing ME reporting. A method to impart information on reporting systems for busy healthcare professionals with hectic schedules is vital to improve ME reporting.

Underreporting of medication errors

In this survey, less than half (44.8%) of the respondents who had encountered ME eventually reported them. Similarly, the actual reporting rates for healthcare professionals are low in practice, resulting in underreporting [14, 23]. This was consistent with audit-based results that found reporting rates as low as 0.12% to 29.2% [24].

In general, pharmacists are more likely to report ME compared to doctors. Pharmacists are trained to look for ME and various training programmes are provided by the Pharmacy Division of the Ministry of Health of Malaysia. In various qualitative studies, pharmacists mentioned that their professional call of duty was to report ME [25, 26] while doctors were reputed not to report them [27].

Hindrances in reporting medication errors

In this study, hindrances to ME reporting were categorised into three factors: hectic work environment, fear of negative outcomes, and the MER-S itself. Common reasons for underreporting as cited by many studies were fear factors, lack of feedback regarding reported ME, and insufficient education on ME reporting [8, 22, 25]. Specifically, the fear of reporting ME faced by healthcare professionals was due to possible impending litigation and other punitive actions by governing boards or the facilities’ management and losing good working relationships with colleagues. Other reasons for not reporting were the laborious process, busy and hectic work environments [8, 23, 25, 28], and the fact that certain ME were perceived as being not harmful [28]. Years of service did not affect the ME reporting practice among the healthcare providers in this survey, echoing the same outcomes suggested by a survey conducted in Manila, the Philippines [15]. Therefore, the mechanisms deducted to increase reporting by healthcare respondents were education on reporting, providing regular feedback, positive changes in the MER-S, electronic reporting formats, and simple reporting methods. Information technology, such as web-based and email reporting, has been suggested by healthcare professionals [29]. However, implementing a reporting system without adequate analysis and feedback on reports did not support reporting and learning [30]. It is necessary to incorporate information technology in ME reporting systems to increase ME reporting for learning through feedback.

Acceptance of smartphone medication error reporting

The healthcare professionals in this survey used their smartphone app for work frequently, which was also reported by other studies [16]. A system such as an anonymous smartphone ME reporting application was an acceptable method for ME reporting among the surveyed doctors and pharmacists. This method of reporting would encourage healthcare professional to report at the point of error. The primary concern for most of these doctors and pharmacists was that the smartphone application should be user-friendly, fast, and feedback enabled. To date, no research has been done on reporting ME using smartphone applications. The study most similar to the present review involved research on a hand-held computer-based application that was well accepted by doctors and nurses [12]. The two substantial challenges in developing such an application would be to ensure the confidentiality of the reporters and to protect secured data storage. The application can be used to educate many healthcare professionals on the trends in ME occurrences and recommend stringent practices with regards to patient safety. Nevertheless, whether the traditional paper method of ME reporting or digital ME reporting, a holistic approach to training for reporting ME is very essential for all healthcare professionals.

Limitations

There were several limitations in this study. The response rate from doctors and pharmacists was poor despite the provision of up to three reminders. The actual number of doctors and pharmacists who received the questionnaire could not be accounted for as the link could have been forwarded to colleagues other than the primary distributor. The primary distributor of the questionnaire was unable to account for the dissemination of the questionnaires via forwarding. Therefore, the actual response rate could be lower than reported here. The reporting rate was lower compared to another study conducted in Malaysia using an email survey form in which a response rate of 49.8% was reported among pharmacists in three Malaysian states [17]. Hence, future studies using this method of questionnaire distribution should be carefully considered in order to retrieve valid and accurate response rates.

A sample selection bias also resulted due to the survey distribution method because the respondents owned a smartphone, had Internet access at work, and were technologically inclined. Finally, the sampling was not stratified based on the proportion of staff in each profession and also in different work settings such as hospitals and clinics. Therefore, the sampling bias among the doctors and pharmacists could have influenced the outcomes and results of this study.

## Conclusions

Underreporting of ME remains prevalent despite continuous efforts to promote the utilisation of local ME reporting systems. Doctors and pharmacists in busy and hectic work environments who have positive responses to ME reporting should be encouraged to utilise the MER-S via a smartphone application should one be available. Hence, creative ways to improve ME reporting and speedy feedback methods such as an anonymous smartphone application should be considered to increase the utilisation of the MER-S in state-funded healthcare facilities.

## Appendices

**Table 5:** Questionnaire used in this survey.

A Survey of Medication Error Reporting and the Acceptability of Smartphone Application Reporting
Section 1: Demographics
Hospital/Clinic Setting: Public Hospital Public Clinic Private Hospital Private Clinic Community Pharmacy Profession Consultant Specialist Medical Officer Pharmacist Work Setting (please indicate discipline, e.g., Medical, Surgery, Community Pharmacy) Years in Service ___________ Age in Years ___________ Gender Male Female
Section 2: Exploring Smartphone Use at Workplace *Medical apps are software applications on smartphones that offer medical information/data that assist in patient care. This includes applications that offer drug/disease databases, medical allow patients to send medical data to healthcare professional, etc., calculators, keep e-medical records, offer medical training, tools for diagnostic examination, allow patients to send medical data to healthcare professional, etc.*
What type of phone do you own? iPhone Android Window Blackberry Others (please specify here) Have you downloaded medical app(s)* to aid your work? Yes No How often do you use your medical app(s) at work? Daily Weekly Monthly Rarely Never How many medical app(s) are available on your smartphone? State the AMOUNT HERE ____________________ State UP TO THREE medical apps that you frequently use Epocrates Medscape Medscape Micromedex Lexicomp Qx Calculate MedCalc SkyScape UpToDate My Blue Book MIMs Others (please specify here) How do you connect to the Internet using your smartphone at work? No Access Employer-Provided Wi-Fi/Hotspot only Employer-Provided Wi-Fi & Personal Data Plan Employer-Funded Data Plan Personal Data Plan Alternative Funded Wi-Fi or Data Plan How would you rate your smartphone’s internet connection at work? Fast Good Average Slow Hardly Able to Connect
Section 3: Exploring Medication Error (ME) Reporting Practice of Healthcare Professionals.
Have you heard of the national medication error reporting system (MERS)? Yes No Have you reported medication error (ME) using MERS? Yes No Have you received reports or statistics of reported MEs? Yes No At your workplace, how often do you encounter ME? Daily Weekly Monthly A Few Times per Year Rarely Not aware of any ME In your opinion, how would you rate the management in handling MEs detected at your workplace? Excellent Good Average Poor Not Sure What is Done Would you report ME that was detected before reaching patient? Yes No In case an ME has reached patient (consumed by/administered to patients), would you report the ME that Did not cause harm? Yes No Caused minor harm to patient (e.g.: requiring additional monitoring/stay)? Yes No Caused serious harm (e.g.: hospitalisation)? Yes No Caused permanent? Yes No Can you give ONE main reason that hinders you from reporting medication errors? Legal Implication (Litigation) Worried about Victimization Impairs Reputation No Time/Too busy Concerns with Reporting Error Committed by Colleague Others (please specify here) Would you report ME via a smartphone application if one is available? Yes No What features of a smartphone application would deem important to you to report ME? (more than one answer allowed) Anonymity/Confidentiality of Identity User-friendly Limited Data Required for the Report Others (please specify here)
